# A multi-layered values-based approach to advance social-ecological restoration: Insights from real-world laboratories in Germany

**DOI:** 10.1007/s13280-025-02259-w

**Published:** 2025-10-11

**Authors:** Konrad Gray, Jacqueline Loos, Berta Martín-López, Maraja Riechers, Anita Kirmer, Miguel Á. Cebrián-Piqueras

**Affiliations:** 1 Department of Agriculture, Ecotrophology, and Landscape Development, Anhalt University of Applied Sciences, Strenzfelder Allee 28, 06406 Bernburg (Saale), Germany; 2 Social-Ecological Systems Institute, School of Sustainability, Leuphana University Lüneburg, 21335 Lüneburg, Germany; 3 Department of Botany and Biodiversity Research, University of Vienna, Rennweg 14, 1030 Vienna, Austria; 4 Institute of Baltic Sea Fisheries, Thünen Institute, Alter Hafen Süd 2, 18069 Rostock, Germany; 5 Kassel Institute for Sustainability, University of Kassel, 34109 Kassel, Germany

**Keywords:** Grassland restoration, Inclusive restoration, Plural values of nature, Social-ecological perspectives, Stewardship, Transdisciplinarity

## Abstract

Calls for social-ecological restoration have emerged to help transcend traditional ecological boundaries by including plural values as well as transdisciplinary processes. Inspired by the IPBES values typology, we used questionnaires to assess four multi-layered perspectives characterizing local actors in two transdisciplinary grassland restoration projects in Germany: Broad values, specific values, knowledge, and visions for restoration outcomes. Varimax rotated principal component analysis yielded 16 factors across these dimensions. A subsequent hierarchical cluster analysis revealed three clusters based on these factors compatible with the Life Framework of Values: *restoration for…* (1) *living with nature,* (2) *living in nature*, and (3) *living from nature*. The three clusters demonstrate the diversity of values-based perspectives among participants of the real-world laboratories. These perspectives capture the multi-layered values underpinning engagement in restoration. Accounting for these perspectives without jeopardizing one perspective over the others provides a basis for more inclusive restoration efforts.

**Supplementary Information:**

The online version contains supplementary material available at 10.1007/s13280-025-02259-w.

## Introduction

Social-ecological systems approaches have gained traction in recent years (Berkes and Folke 1994; Ostrom 2009; Manyani et al. 2024), advancing many research fields, such as conservation biology (Ban et al. 2013; Palomo et al. 2014), agroecology (Lescourret et al. 2015) and restoration ecology (Martín-López and Montes 2015; Fischer et al. 2021). This approach increasingly moves into the practice of ecological restoration (Fernández-Manjarrés et al. 2018). Ecological restoration refers to “the process of assisting the recovery of an ecosystem that has been degraded, damaged, or destroyed” (Gann et al. 2019, p. 7). Calls to transform ecological restoration into a social-ecological endeavor emphasize the importance to recognize human dimensions of restoration beyond ecological dimensions (Fischer et al. 2021; Tedesco et al. 2023). However, to date, these have been only marginally included in restoration projects (Sigman and Elias 2021; Elias et al. 2022).

One benefit of a social-ecological systems approach for restoration (henceforth referred to as social-ecological restoration) is acknowledging that people can be stewards (Fischer et al. 2021). Transdisciplinary and participatory processes offer a potential way to help reconnect people to nature, foster engagement and cultivate stewardship (Folke et al. 2011; Ives et al. 2017; Fox and Cundill 2018; Bieling et al. 2020; Fischer et al. 2021). To better understand how people can act as stewards and engage in social-ecological restoration, there is a need to explore people’s values towards nature, their knowledge, and visions of desirable futures (Bieling et al. 2020; Gottwald and Stedman 2020). Underlying values and visions that motivate environmental stewardship and engagement are not yet well understood (Mikołajczak et al. 2023), particularly in restoration practices. Investigating plural values and visions offers an understanding of the foundation for the engagement of actors with diverse perspectives on restoration.

Plural values of nature have lately received increasing attention in environmental management and decision-making (Arias-Arévalo et al. 2017; Zafra-Calvo et al. 2020; Schmitt et al. 2022; Pascual et al. 2023) thereby counteracting the historical dominance of monetary and instrumental values in scientific literature (Gómez-Baggethun et al. 2010; Thorén and Stålhammar 2018; IPBES 2022; Gross et al. 2023). The recognition that people value nature in multiple ways is crucial for effectively managing social-ecological systems and restoring people’s capacity to manage nature sustainably (Baveye et al. 2013; Gómez-Baggethun and Martín-López 2015; Jacobs et al. 2018). The benefits of plural valuation include helping to resolve conflicts by managing and communicating trade-offs, adding transparency and justification to decision-making, and fostering public support through potential stakeholder engagement (Ives and Kendal 2014; Jones et al. 2016).

Drawing on extensive research on values across disciplines (Rokeach 1973; Schwartz 1992; Gibson and Koontz 1998; Vining et al. 2000), the Intergovernmental Science-Policy Platform on Biodiversity and Ecosystem Services (IPBES) has advanced a value typology which acknowledges plural values of nature. This typology recognizes the interplay of plural values across multiple layers, distinguishing worldviews and knowledge systems, broad values, specific values, and value indicators (IPBES 2022). Worldviews and knowledge systems describe how people perceive their surroundings and interpret the world (Pascual et al. 2023), which affects people’s material and non-tangible relations with nature. For example, Cebrián-Piqueras et al. (2020) examined ecological knowledge and its role in shaping perceptions of protected areas, providing insights into the interdependent knowledge systems that influence human-nature relationships of local communities and highlighting inclusive approaches accounting for the plurality of worldviews for sustainable landscape management. Broad values relate to guiding life principles (Schwartz 1992; Manfredo et al. 2017; Kendal and Raymond 2019; Goodson et al. 2023; Pascual et al. 2023). These broad values have been identified as essential levers for transformative change (Martin et al. 2022; Harmáčková et al. 2023), yet they are difficult to target (Manfredo et al. 2017; Fischer 2020; Andrade et al. 2023). Goodson et al. (2023) investigated the role of broad values in managing protected areas. They found that these values potentially guide deliberation by affecting discussion topics, thereby highlighting their importance in achieving common ground for inclusive conservation. Yet, broad values remain underexplored when designing and developing restoration projects. Specific values refer to the importance, worth, or usefulness attributed to nature in particular contexts, ecosystems, or natural entities (Pascual et al. 2023). Specific values are categorized as intrinsic, instrumental and relational values (Muraca 2011; Chan et al. 2016). Although all three value categories are relevant for the design of inclusive conservation measures (Himes and Muraca 2018; Lo et al. 2022), for mitigating social conflicts, and enhancing communication among different groups (Arias-Arévalo et al. 2017; Gale and Ednie 2020), the consideration of relational values remains sparsely explored (Himes et al. 2024), particularly in restoration literature (Wainaina et al. 2023). Moreover, studies applying a multi-layered values approach that considers both broad and specific values to inform conservation and restoration practices remain scarce (Harmáčková et al. 2023; Cebrián-Piqueras et al. 2025).

People´s values of nature and human-nature relationships influence differing opinions on ecological restoration methods (Hertog and Turnhout 2018). Both broad and specific values underpin human actions that, in turn, can impact the restoration process and alter developments (Kibler et al. 2018). Inspired by the IPBES values typology, we adapted the multi-layered values proposition to a restoration context by investigating both broad and specific values alongside knowledge for restoration and desired restoration outcomes (i.e., visions for restoration). For our context, we substituted the IPBES knowledge system and worldview level with the more applicable approaches to knowledge types and visions for restoration.

Based on the assumption that values, knowledge, and visions are interdependent (Horcea-Milcu et al. 2022), we expected that values concerning self-enhancement (i.e., egoistic values) and instrumental values would cluster more with knowledge on forage productivity (i.e., knowledge about the generation of biomass and fodder in grassland ecosystems) and visions of restoring ecosystems for the purpose of enhancing their capacities to provide specific or short-term benefits (Stern et al. 1999; de Groot and Steg 2008; Goodson et al. 2023; Cebrián-Piqueras et al. 2025). Conversely, more intrinsic, biospheric values and knowledge on ecological functioning presumably cluster with more historical ecological restoration visions that aim to recover the naturalness of ecosystems. Identifying clusters through this multi-layered approach has the potential to enhance an understanding of the local social-ecological context. This understanding can facilitate the design of restoration interventions that cultivate stewardship tailored to the local actors’ values, knowledge, and visions (Bennett et al. 2018).

Restoring grassland ecosystems is crucial for halting and reversing biodiversity decline (Petermann and Buzhdygan 2021; Török et al. 2021; Staude et al. 2023). Besides being among the most threatened ecosystems (Hoekstra et al. 2004), species-rich grasslands harbor a great part of the Central European plant diversity (Dengler et al. 2014). Grasslands are vital habitats for many rare plants, pollinators and other flower-visiting insects (Wilson et al. 2012; Petermann and Buzhdygan 2021), a basis for complex multitrophic interactions (Rzanny and Voigt 2012), and contribute to people’s quality of life in multiple ways. Nature’s contributions to people (NCP) are defined as all contributions that people derive from nature (Díaz et al. 2018). NCP explicitly includes ecosystem services, nature’s gifts and other analogous concepts (Hill et al. 2021; IPBES 2022). Apart from the material and regulating NCP, which often reflect instrumental values (Pascual et al. 2017), grasslands also supply non-material NCP, such as opportunities for tourism and recreation (Bengtsson et al. 2019) and therewith represent places to nurture relationships with nature and other people, reflecting relational values (Schmitt et al. 2022). Even though grassland species are declining (Wepprich et al. 2019; Warren et al. 2021; Jandt et al. 2022), grasslands are often underrepresented in global restoration policies (Veldman et al. 2019), and the NCP provision is often underappreciated (Bengtsson et al. 2019; Temperton et al. 2019; Staude et al. 2023). Grasslands are important ecosystems that typically rely on sustainable human use as their main threats are agricultural intensification and land abandonment (Dengler et al. 2014). Thus, restoration ecologists, for example, are investigating options for balancing farmers’ biomass production needs with nature conservation goals (Dullau et al. 2023).

To advance values-based social-ecological restoration, we established real-world laboratories in the context of grassland restoration in Germany. A real-world laboratory conceptualizes a transdisciplinary approach for transformative research focusing on a process-oriented, long-term, embodied experience within a reflexive learning environment and grounded in real-world contexts (Schäpke et al. 2018). Hence, social-ecological restoration through a real-world laboratory can reconnect humans to their environments and empower them as stewards in navigating restoration processes. For this study, we aimed to elicit people’s values and knowledge of grassland, as well as visions for grassland restoration prior to a transdisciplinary restoration process. We explored what people value about grassland, examining not only the relationship between broad and specific values, but extending the relations of these values with knowledge and visions in the restoration context. Consequently, we employed a multi-layered values approach, following the IPBES values typology, substituting knowledge systems and worldviews with knowledge of and visions for grassland restoration, to advance social-ecological restoration. Through this multi-layered assessment of broad values, specific values, knowledge and visions for grassland restoration, we identified clusters that reveal three different social-ecological restoration perspectives among the real-world laboratory participants. Finally, we discuss how these perspectives provide a basis for inclusive (i.e., equitable) restoration that can contribute to cultivating stewardship among different social actors.

## Materials and methods

### A transdisciplinary approach to grassland restoration

This study was conducted during the initial phase of the inter- and transdisciplinary project *Grassworks* that investigated success factors for grassland restoration in Germany (Temperton et al. 2025). Part of the project involved establishing real-world laboratories through which we collaboratively planned, implemented and evaluated grassland restoration measures together with local actors. Following a transdisciplinary research mode, the real-world laboratories served as spaces for knowledge co-creation, experimentation, learning and reflexivity. By implementing the real-world laboratories, we aimed to activate transformative and long-term oriented processes that are suitable for scalability and transferability (Schäpke et al. 2018).

**Fig. 1:**
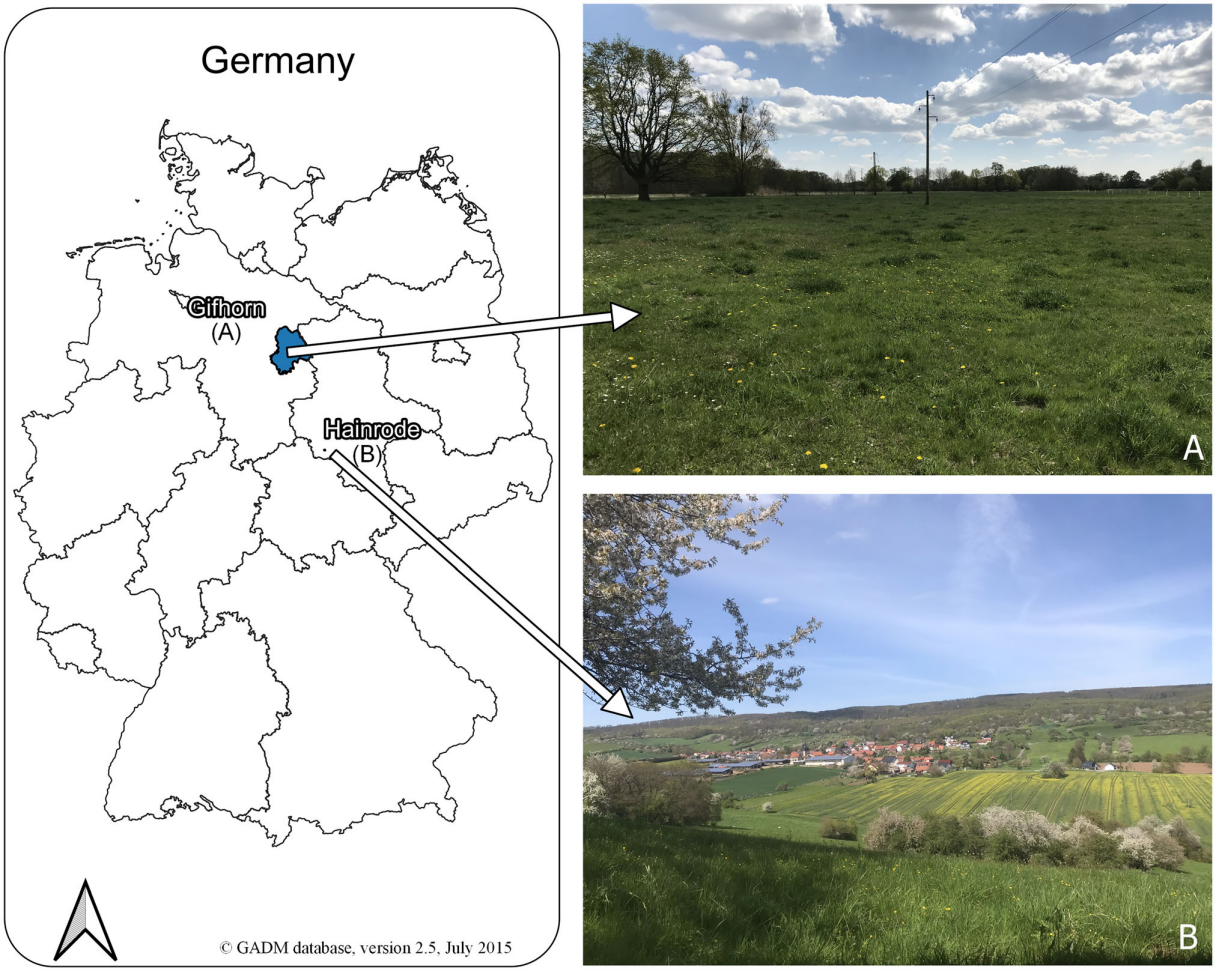
Location of the two real-world laboratories for grassland restoration in Germany. **A** Northern real-world laboratory with a focus on the county of Gifhorn. **B** Real-world laboratory in the center of Germany with a focus on the village of Hainrode. Photos: Corresponding author

For our analysis, we focused on two real-world laboratories: (A) Gifhorn and (B) Südharz. One real-world laboratory (A) is situated in the northern part of Germany in the county of Gifhorn (Fig. 1). The main contacts are with a local nature conservation NGO, the administrative office of Gifhorn, and farmers from the area. The social-ecological boundaries are defined by the county of Gifhorn, and the potential restoration intervention focuses on large-scale agricultural landscapes, with local farmers expected to be the main participants in the co-design and development of restoration measures.

The other real-world laboratory (B) is situated within the biosphere reserve *Karstlandschaft Südharz [Karst landscape South Harz]*, in the southern Harz region (Fig. 1). The center of the real-world laboratory is the village of Hainrode, whose inhabitants form the basis of the transdisciplinary collaboration. The Hainrode local history and nature conservation association is the primary contact, leveraging their strong village network to organize various local activities and reach potential participants. Potential restoration interventions emphasize communicating biodiversity measurements and enhancing restoration acceptance, aligning with the association’s interests.

### Survey design and items

We designed a survey that included, among other items, item subsets focusing on different multi-layered individual dimensions concerning human-nature relationships: i.e., broad values, (Table 1), specific values (Table 2), knowledge (Table 3), and potential desired goals of restoration activities (i.e., visions) (Table 4). We used a 4-point Likert scale for each subset (1—Very unimportant, 2—Rather unimportant, 3—Rather important, 4—Very important). For the knowledge subset we used a 3-point Likert scale (1—low knowledge, 2 – medium knowledge, 3—high knowledge), which was adapted from D’Antonio et al. (2012) to assess self-rated knowledge of participants (Table 3). We pre-tested the survey with members of the project´s research consortium and people unrelated to the project, but having knowledge about grasslands, and adapted it accordingly.

**Table 1:** The broad values subset with the broad value types, survey statements, answer scale and references. Survey statements adapted from de Groot and Steg (2008), Winkler-Schor et al. (2020), Shin et al. (2022), Andrade et al. (2023), and Goodson et al. (2023). (Answer scale: 1—Very unimportant; 2—Rather unimportant; 3—Rather important; 4—Very important)

Broad value types	Survey statements (*Please rate the importance of the following values as guiding principles in your life*)
Altruistic	Social justice: correcting injustice, care for others all
	Equality: equal opportunity for all
	A world at peace: free of war and conflict
Biospheric	A world of beauty: beauty of nature and the arts
	Unity with nature: fitting into nature
	Protecting the environment: preserving nature
Egoistic	Authority: the right to lead or command
	Influential: having an impact on people and events
	Social power: control over others, dominance
Eudaimonic	Personal growth: development of new skills, learning, or gaining insight into something
	Pursuit of excellence: attaining a personal ideal in life
	Autonomy: deciding your own future and doing what you believe
	Satisfaction with life: finding meaning, value, and relevance to a broader context
Hedonistic	Fulfillment of desire: food, fun, pleasure
	Enjoying life: pursuing hobbies, leisure, socializing
	Reducing anxiety: seeking comfort and relaxation

**Table 2:** The specific values subset with the specific value types, value categories and survey statements. Survey statements adapted from Arias-Arévalo et al. (2017), Klain et al. (2017), Uehara et al. (2020), Inglis and Pascual (2021), Liu et al. (2022), Pellaton et al. (2022), Schmitt et al. (2022), Saito et al. (2022), and Riechers et al. (2022). (Answer scale: 1—I do not agree; 2—I rather disagree; 3—I rather agree; 4—I completely agree). *nuanced expressions of sense of place

Specific value types	Value categories	Survey statements (*I value grassland in my region because…*)
Instrumental	Regulating	…it provides us with clean water and soil fertility
	Economic	…I economically benefit from it
	Provisioning	…it provides fodder and hay
Intrinsic	Grasslands´ inherent reason of existence	…I recognize that grassland in my region has its particular reason for its existence, and therefore, it is worth of conservation
	Species´ inherent right to exist	… grassland species in my region have a right to exist
	Grasslands rights to exist and thrive	…it has its own right to exist and prosper
Relational	Cultural identity	…it is an important part of our culture
	Individual identity	…it is an important part of who I am
	Learning	…I learn through grassland about myself
	Collective identity	…it connects me with who we are as a community
	Cultural heritage	…it is a place of heritage and history that is important for me, our community, and the region
	Traditions	…it is a place for our traditions and the way of life of my ancestors
	Community support	…it supports other people in my community
	Social relations	…it gives me the opportunity to have relationships with friends, family, and other people
	Place connection*	…being here fosters a sense of belonging
	Feeling home*	…it contributes to a feeling of home
	Care	…my care for grassland helps me to have a good and fulfilling life
	Stewardship	…I feel responsible to protect from negative impacts
	Aesthetics	…I enjoy the beauty of the scenery, sounds, and smells
	Inspiration	…it inspires me with new ideas and creativity
	Therapeutic	…it makes me feel better, physically and/or mentally
	Relaxation	…it has a calming and relaxing effect on me

**Table 3:** The knowledge subset with the knowledge themes and survey statements. References cf. Bardgett et al. (2021), D’Antonio et al. (2012), Dullau et al. (2019), BfN/BMU (2019), Lyons et al. (2023). (Answer scale:1—low knowledge, 2—medium knowledge, 3—high knowledge)

Knowledge theme	Survey statements (*Please rate your level of knowledge on the following topics relating to grassland*)
Soil	Soil fertility
Water	Water quality and quantity
Plants	Plant diversity
Management	Management / cultivation
Animals	Animal diversity
Grassland Maintenance	Grassland maintenance
Restoration of species-rich grasslands	Restoration of species-rich grasslands
Financing	Financing
Traditional practice	Traditional crafts / traditional techniques (e.g. scythe mowing)

**Table 4:** The visions subset with the (sub-) categories and survey statements following the representation of the ecological recovery and social benefits wheel from Gann et al. (2019). (Answer scale: 1—I do not agree; 2—I rather disagree; 3—I rather agree; 4—I completely agree)

Category	Sub-category	Survey statements (*In my opinion, the successful restoration of species-rich grassland has the goal of…*)
Ecological	Ecosystem functions ensured	…ensuring ecological functions, such as nutrient or water cycles
	Absence of threats	…mitigating negative influences on biodiversity, such as over-fertilization, land abandonment, or invasive species
	Species composition balanced	…balancing the species composition in grassland, i.e., ensuring the presence of native species and absence of invasive species
	Ecosystem and species integrated with the landscape	…appropriately integrating the species-rich grassland into its wider landscape, so that, for example, exchange between animals and plants in different habitats is possible
	Structural diversity	…ensuring structural diversity, e.g., diverse food chains and habitat diversity
	Environmental conditions maintained	…maintaining environmental conditions, e.g., nutrient content in the soil or climatic conditions, which are necessary for the preservation of species richness
Social	Stakeholder engagement	…maintaining stakeholder commitment, so that, for example, new stakeholders become involved or the biodiversity continues to be supported
	Health and well-being	…improving the quality of life for people in the surrounding area (e.g., as a contribution to promoting relaxation and well-being)
	Restoring natural capital (ecosystem services)	…restoring and preserving natural assets and ecosystem services, such as carbon storage and soil fertility
	Knowledge enrichment	…generating new knowledge and reinforcing traditional knowledge, e.g., through innovative projects, research, and exchange with practitioners
	Sustainable economies	…maintaining sustainable economic practices and profitability, e.g., through the creation and safeguarding of jobs
	Benefits distribution	…fairly distributing the benefits and useful services of species-rich grassland

#### Broad values

The broad values subset consisted of 16 variables representing five broad value types: altruistic (3 statements), biospheric (3 statements), egoistic (3 statements), eudaimonic (4 statements), and hedonistic (3 statements) values (Table 1). Survey statements for this subset were adapted from de Groot and Steg (2008), Winkler-Schor et al. (2020), Shin et al. (2022), Goodson et al. (2023), and Andrade et al. (2023).

#### Specific values

The specific values subset represented the three types of values, including intrinsic (3 statements), instrumental (3 statements), and relational values (16 statements). Since relational values cover a range of different aspects, they represent the largest value sub-category that can be divided into smaller nuanced types such as cultural identity, individual identity, cultural heritage, traditions, sense of place, inspiration, and relaxation, among others (Table 2). The survey statements for the specific values were adapted from previous scholarly work to fit our particular case (Arias-Arévalo et al. 2017; Klain et al. 2017; Uehara et al. 2020; Inglis and Pascual 2021; Liu et al. 2022; Pellaton et al. 2022; Riechers et al. 2022; Saito et al. 2022; Schmitt et al. 2022).

#### Knowledge

The knowledge subset was designed around knowledge themes that are relevant for grassland ecosystem restoration developed through the experience of the authors and discussions with experts from the *Grassworks* project (cf. D’Antonio et al. 2012; Dullau et al. 2019; BfN/BMU 2019; Bardgett et al. 2021; Lyons et al. 2023). This subset covers 9 themes all relating to different aspects of grasslands, including soil, water, plants, management, animals, maintenance, restoration, financing, and traditional practices (Table 3).

#### Visions

**Fig. 2:**
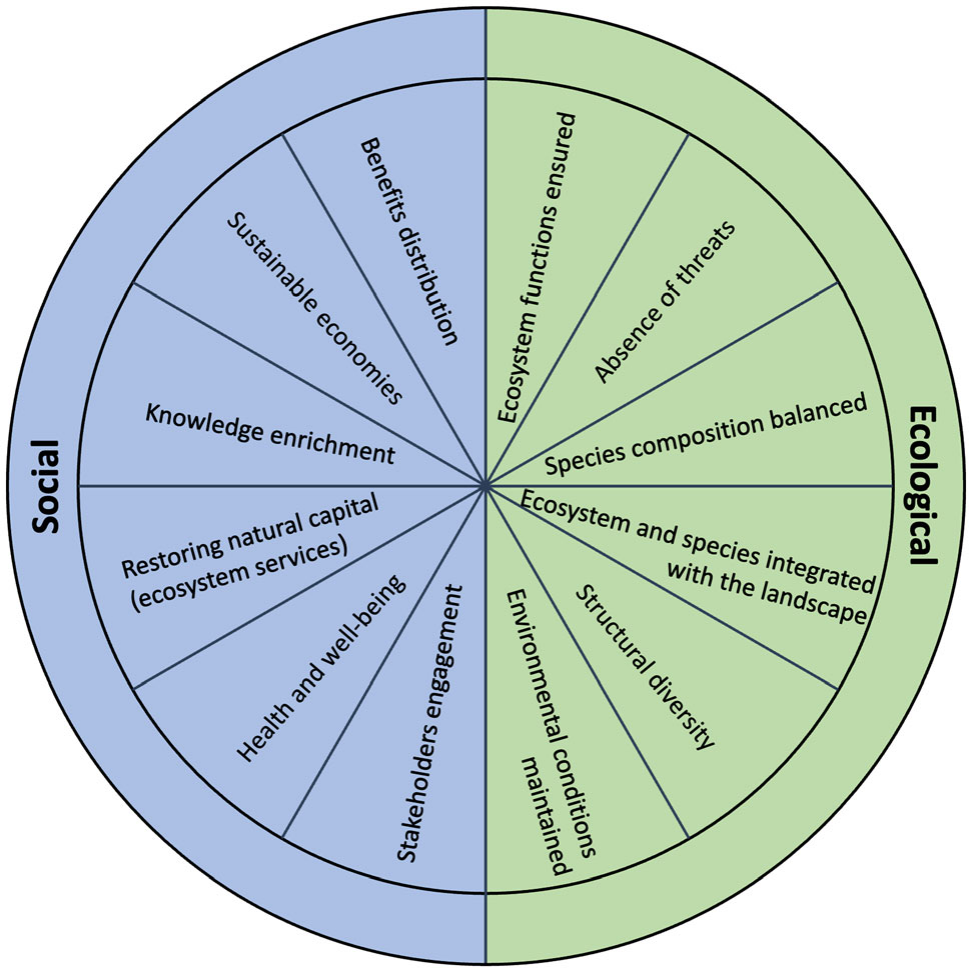
Simplified representation of the ecological recovery and social benefits wheel from Gann et al. (2019). *Source* Own design

The visions’ subset was derived from the ecological recovery and social benefits wheels from the Society for Ecological Restoration (Gann et al. 2019). These wheels are design tools to track restoration goals and can be specifically adapted to individual restoration projects. However, as the study was conducted before the restoration projects in the real-world laboratories started, we decided to adapt the examples provided by Gann et al. (2019) and not design targeted potential goals ourselves that could bias the process of the real-world laboratories. Thus, the survey statements for visions of restoration outcomes relate to six social benefits, as well as six ecological benefits, i.e., improvements for ecosystem conditions (Table 4 and Fig. 2).

### Data collection

The sample population consisted of the participants of the real-world laboratories who attended the first workshop or were referred to us through our main contacts at the time of data collection. Therefore, we consider our sample size representative of the real-world laboratories’ population at that time. The dynamic transdisciplinary processes can affect the composition of the participants in the real-world laboratories, meaning that key actors may change throughout the project. The survey was distributed at the initial stage of the transdisciplinary process in order to collect self-reported data prior to the influence of the project (see Methodological Limitations). A paper survey was administered at the initiating workshops in each real-world laboratory in February 2023 (n = 29), and subsequently at individual encounters with stakeholders on-site (n = 8). In addition, an online survey was distributed after the first workshop to reach people who could not participate. This survey was circulated until the beginning of August 2023 and gathered 13 responses (completion rate 53.6%). In total, we reached n = 50 participants (real-world laboratory Gifhorn (A) with n = 29; real-world laboratory Hainrode (B) with n = 21).

### Analysis

In our explorative study, we assessed the multi-layered values for social-ecological restoration through varimax rotated principal component analysis (varimax rotated PCA), followed by a hierarchical cluster analysis with the extracted varimax factors using R and RStudio (Posit team 2023; R Core Team 2023). This two-step approach allowed us to handle the complexity of the data while retaining most of the information (varimax rotated PCA) and yet exploring the relations and structure of the multi-layered values (hierarchical cluster analysis). Prior to the multivariate analysis, we replaced missing values using the k-nearest neighbor method (k = 5) from the R package VIM (Kowarik and Templ 2016). Further, we tested each subset for suitability for the PCA with the Kaiser–Meyer–Olkin (KMO) measure and Bartlett’s test. The KMO is a measure of sampling adequacy by comparing the correlations between variables to the partial correlations. Each subset resulted in an overall KMO ≥ 0.75, indicating good suitability according to Field et al. (2012). We used Bartlett’s test to evaluate whether the correlation matrix significantly differs from an identity matrix (Field et al. 2012). Since all subsets were tested with a *p*-value < 0.05, correlations between items were sufficiently significant for PCA (Supplementary Information). Therefore, we proceeded with a PCA for each subset to reduce dimensions. We used the principal function from the psych package with the rotation parameter set to “none” and applied the Spearman method for the correlation as we handled non-parametric data (William Revelle 2023). We rounded the eigenvalues to one decimal place to extract relevant components and applied the Kaiser Criterion (eigenvalue > 1). However, for the knowledge subset, we also included the third component, which had an eigenvalue of 0.9, to accomplish a cumulative variance of over 60%. Subsequently, we applied a varimax rotation for each subset with the extracted components, using the principal function again to produce more interpretable results (resulting in varimax factors). All variable loadings above 0.4 after rotation were regarded as a relevant variable of the varimax factors (Stevens 2009; Field et al. 2012; Shrestha 2021). The resulting scores from the varimax factors were then used in a hierarchical clustering with the R native hclust function applying Euclidean distance and using Wards method (R Core Team 2023).

### Methodological limitations

Our study is subject to caveats. The survey was designed to assess local actors from the two real-world laboratories. Thus, the results should only be interpreted within their social-ecological boundaries. The clusters represent value-based social-ecological restoration perspectives from within the real-world laboratories, and we caution over-interpreting them to represent a general depiction of values-based social-ecological restoration. Due to the limitations of the sample size and the amalgamation of two real-world laboratories, it is not feasible to directly assign which cluster prevails in which real-world laboratory. Additionally, the varimax factors have limitations due to the small sample size, which may lead to overfitting and affect factor stability. Furthermore, our survey was distributed early in the transdisciplinary process to gather unbiased responses, with minimal interactions between researchers and participants until survey completion. While we value qualitative and participatory methods for eliciting social-ecological complexity, we intentionally minimized engagement at the start of the real-world laboratories to prevent influencing results and to keep their development flexible.

## Results

### Broad values

The varimax-rotated PCA resulted in five varimax factors, which explain 65.6% of the variance in the broad value subset. Table 5 illustrates the composition of the five varimax factors (henceforth: factors) with the factor loadings after rotation for each value, as well as the descriptive properties of each item. We labeled the first factor (19.40% of the variance) as “nurturing the world for harmony” as its components suggest a focus on mainly altruistic (i.e., *social justice, equality, a world at peace*) and biospheric values (i.e., *unity with nature, and protection of the environment*) with an addition of the eudaimonic value of *life satisfaction*. The second factor (12.93%) with eudaimonic values as *personal growth* and *autonomy,* as well as the one addition of *need satisfaction* (hedonistic), suggested a valuation of “independence for personal development”. “Leading for the better” is our label for the third factor (12.28%), which represents the values of *authority* and *social power* (egoistic) as well as *striving for excellence* (eudaimonic) and *need satisfaction* (hedonistic). The fourth factor (11.58%) consisted of hedonistic and biospheric values. With a *world of beauty* and *unity with nature,* as well as *reducing anxiety* and *enjoying life,* it corresponded with the notion of pursuing an “unencumbered life in harmony with nature”. We labeled the last factor (9.44%) as “impactful living with intention” as it mainly consisted of egoistic values such as *influential* and *social power* with an influence of *a world of beauty* (biospheric).

**Table 5:** Factor loadings from the varimax-rotated PCA for the subset of broad values. Factor loadings above 0.4 are shown in bold. (M = mean; SD = Standard Deviation)

Type	Broad value	Descriptive		Varimax factors				
		*M*	*SD*	F1: Nurturing the world for harmony	F2: Independence for personal development	F3: Leading for the better	F4: Unencumbered life in harmony with nature	F5: Impactful living with intention
Altruistic	Social justice	3.50	0.71	**0.578**	0.332	− 0.143	0.138	− 0.030
	Equality	3.48	0.76	**0.669**	0.226	− 0.109	0.079	− 0.105
	A world at peace	3.62	0.70	**0.749**	0.019	0.045	0.114	− 0.110
Biospheric	A world of beauty	3.14	0.70	0.364	0.272	− 0.082	**0.496**	**0.452**
	Unity with nature	3.32	0.62	**0.524**	− 0.117	− 0.031	**0.481**	0.311
	Protection of the environment	3.74	0.56	**0.792**	− 0.061	− 0.154	0.072	0.048
Egoistic	Authority	1.78	0.62	− 0.254	− 0.091	**0.690**	0.150	0.247
	Influential	2.26	0.72	− 0.058	0.152	0.063	− 0.053	**0.826**
	Social power	1.38	0.49	− 0.153	− 0.080	**0.551**	0.146	**0.556**
Eudaimonic	Personal growth	3.36	0.78	0.306	**0.765**	− 0.076	0.090	0.105
	Striving for excellence	2.46	0.76	0.098	0.239	**0.829**	− 0.012	− 0.118
	Autonomy	3.28	0.73	0.032	**0.810**	0.139	0.110	0.070
	Life satisfaction	3.46	0.73	**0.688**	0.252	0.303	− 0.133	0.099
Hedonistic	Reducing anxiety	2.90	0.84	0.238	0.321	0.324	**0.641**	0.249
	Need satisfaction	2.88	0.75	0.047	**0.543**	**0.443**	0.316	0.026
	Enjoying life	3.14	0.73	− 0.006	0.144	0.092	**0.858**	− 0.168
Proportional Variance (%)				19.40	12.93	12.28	11.58	9.44
Cumulative Variance (%)				19.40	32.33	44.61	56.19	65.63
Sums of Squared Loadings				3.10	2.07	1.96	1.85	1.51

### Specific values

The varimax-rotated PCA resulted in five varimax factors for the specific values, explaining 70.6% of the variance. The first factor (17.74% of the variance) was composed of only relational values, which mainly represent community aspects. These were *collective identity*, *cultural heritage*, *traditions*, *community support*, *social relations* and *sense of place* (Table 6). However, also a few relational values with more individual properties, such as *inspiration*, *aesthetics* and *learning* were included in the first factor. Consequently, we labeled this factor “cultural continuity”. The second factor (16.47%), which we labeled “thriving together”, constituted a set of relational values as well as the *economic* instrumental value. The relational values represented a variety of individual and collective properties, with *individual identity*, *learning*, *inspiration*, and *feeling home* and *traditions*, *social relations*, and *care*. The third factor (14.44%) was a bundle of instrumental and relational values. Here, the instrumental value referred to *regulating* ecosystem services and the relational values were indicated through *cultural identity*, *feeling home*, *aesthetics*, and well-being aspects such as *therapeutic* and *relaxation*. Thus, we labeled this factor as “well-being through nature’s benefits”. The fourth factor (13.10%), named as “caring for nature’s legacy”, mainly represented the intrinsic values of the grassland ecosystem and grassland species. However, it also embodied the relational value of *stewardship*. We labeled the last factor (8.85%) as “agricultural heritage” because it included mainly instrumental values with *regulating* and *provisioning* ecosystem services, yet also the relational values of *cultural identity* and *traditions*.

**Table 6:** Factor loadings from the varimax-rotated PCA for the subset of specific values. Factor loadings above 0.4 are shown in bold. (M = mean; SD = Standard Deviation). *nuanced expressions of sense of place

Type	Specific value	Descriptive		Varimax factors				
		*M*	*SD*	F1: Cultural continuity	F2: Thriving together	F3: Well-being through nature’s benefits	F4: Caring for nature’s legacy	F5: Agricultural heritage
Instrumental	Regulating (clean water and soil fertility provision)	3.54	0.71	− 0.001	0.298	**0.540**	0.195	**0.467**
	Economic (provisioning—profit from grasslands)	2.32	1.20	0.112	**0.794**	− 0.071	− 0.044	0.323
	Provisioning (food provision—hay and fodder)	3.78	0.42	0.319	0.369	− 0.124	− 0.186	**0.732**
Intrinsic	Grasslands own reason of existence	3.82	0.44	0.235	− 0.072	0.336	**0.706**	− 0.125
	Species own right to exist	3.84	0.47	0.316	− 0.032	0.054	**0.687**	− 0.065
	Grasslands rights to exist and thrive	3.54	0.71	0.102	0.136	0.094	**0.819**	0.094
Relational	Cultural identity	3.68	0.59	0.135	− 0.029	*0.456*	0.326	**0.665**
	Individual identity	2.92	1.03	0.315	**0.694**	0.256	0.212	− 0.135
	Learning	2.46	0.97	**0.592**	**0.591**	0.317	0.112	− 0.013
	Collective identity	2.76	1.00	**0.793**	0.332	0.036	0.158	0.145
	Cultural heritage	3.26	0.90	**0.687**	0.119	0.132	0.378	0.320
	Traditions	3.02	1.00	**0.512**	**0.433**	0.054	0.053	**0.472**
	Community support	2.80	0.93	**0.668**	0.236	0.146	0.243	− 0.247
	Social relations	2.50	1.07	**0.640**	**0.487**	0.180	− 0.031	0.183
	Place connection*	3.28	0.88	**0.654**	0.066	0.242	0.094	0.215
	Feeling home*	3.54	0.71	0.210	**0.425**	**0.550**	0.323	0.013
	Care	2.88	1.02	0.155	**0.764**	0.156	0.120	0.153
	Stewardship	3.56	0.54	− 0.084	0.361	0.105	**0.738**	0.244
	Aesthetics	3.84	0.37	**0.461**	− 0.154	**0.622**	0.275	0.146
	Inspiration	2.98	0.87	**0.414**	**0.573**	0.329	0.078	0.092
	Therapeutic	3.54	0.73	0.068	0.179	**0.842**	0.113	0.008
	Relaxation	3.50	0.76	0.214	0.153	**0.832**	0.043	0.022
Proportional Variance (%)				17.74	16.47	14.44	13.10	8.85
Cumulative Variance (%)				17.74	34.21	48.65	61.75	70.60
Sums of Squared Loadings				3.90	3.62	3.18	2.88	1.95

### Knowledge

For the knowledge subset, the varimax-rotated PCA resulted in three varimax factors which explain 67.2% of the variance. The first factor (27.47% of the variance) covered an extensive part of the environmental knowledge themes, such as *soil*, *water*, *plants*, as well as some technical knowledge, i.e., *grassland maintenance* and *restoration* knowledge. Hence, we labeled this factor “environmental (restoration) knowledge” (Table 7). We labeled the second knowledge factor (27.13%) as “production knowledge”, as it consisted mainly of knowledge themes related to agricultural production such as *management*, *animals*, *maintenance* and *financing* knowledge. The last factor (12.56%) only included the *traditional practice* theme, which is why we labeled it as “traditional knowledge”.

**Table 7:** Factor loadings from the varimax-rotated PCA for the subset of knowledge. Factor loadings above 0.4 are shown in bold. (M = mean; SD = Standard Deviation)

Knowledge theme	Descriptive		Varimax factors		
	*M*	*SD*	F1: Environmental (restoration) knowledge	F2: Production knowledge	F3: Traditional knowledge
Soil	1.84	0.65	**0.775**	0.190	− 0.044
Water	1.72	0.54	**0.637**	0.177	− 0.198
Plants	2.12	0.66	**0.752**	0.200	0.028
Management	2.06	0.68	0.319	**0.708**	0.017
Animals	1.96	0.53	0.218	**0.824**	0.060
Grassland Maintenance	2.00	0.64	**0.475**	**0.681**	0.187
Restoration of species-rich grasslands	1.84	0.68	**0.718**	0.371	0.258
Financing	1.54	0.65	0.099	**0.743**	− 0.376
Traditional practice	1.52	0.58	− 0.026	− 0.013	**0.918**
Proportional variance (%)			27.46	27.13	12.56
Cumulative Variance (%)			27.46	54.59	67.16
Sums of Squared Loadings			2.47	2.44	1.13

### Visions

For the visions’ subset, the varimax-rotated PCA also resulted in three varimax factors, which explain 68.1% of the variance. The first factor (32.10%) included many ecological-oriented outcomes and a few social-oriented outcomes (Table 8). We labeled this factor “ecosystem restoration”, as it focused mainly on ecological recovery aspects, such as *ecosystem functions ensured*, *absence of threats*, *species composition balanced, ecosystem and species integrated with the landscape*, *structural diversity*, and *environmental conditions maintained*. However, with *restoring natural capital (ecosystem services)* and *knowledge enrichment,* it also comprised some social-oriented outcomes. “Restoring well-being”, on the other hand, is the factor (18.31%) which consisted of only social-oriented restoration outcomes, such as *stakeholder engagement*, *ecosystem services*, *knowledge enrichment*, *health and well-being*. The third factor (17.72%) included *environmental conditions maintained* as the ecological outcome and *sustainable economies* and *benefits distribution* as the more social-oriented outcomes. We labeled this visions factor as “restoring thriving grassland”, as it seems to relate to sustainable agricultural practices with ecological integrity.

**Table 8:** Factor loadings from the varimax-rotated PCA for the subset of visions. Factor loadings above 0.4 are shown in bold. ‘Knowledge enrichment’ with a loading of 0.399 was regardless considered for the “ecosystem restoration” factor. (M = mean; SD = Standard Deviation)

Category	Sub-category	Descriptive		Varimax factors		
		*Mean*	*SD*	F1: Ecosystem restoration	F2: Restoring well-being	F3: Restoring thriving grassland
Ecological	Ecosystem functions ensured	3.72	0.54	**0.760**	0.204	0.361
	Absence of threats	3.72	0.57	**0.745**	0.303	0.180
	Species composition balanced	3.62	0.64	**0.699**	0.249	0.109
	Ecosystem and species integrated with the landscape	3.78	0.55	**0.856**	0.157	0.284
	Structural diversity	3.76	0.56	**0.838**	0.086	0.342
	Environmental conditions maintained	3.66	0.63	**0.538**	0.129	**0.609**
Social	Stakeholder engagement	3.56	0.64	0.219	**0.740**	0.241
	Health and well-being	3.24	0.74	0.037	**0.893**	0.190
	Restoring natural capital (ecosystem services)	3.66	0.66	**0.435**	**0.424**	0.178
	Knowledge enrichment	3.48	0.71	**0.399**	**0.620**	-0.088
	Sustainable economies	3.46	0.76	0.177	0.162	**0.818**
	Benefits distribution	3.46	0.76	0.279	0.134	**0.762**
Proportional variance (%)				32.10	18.31	17.72
Cumulative Variance (%)				32.10	50.41	68.13
Sums of Squared Loadings				3.85	2.20	2.13

### Clusters

**Fig. 3:**
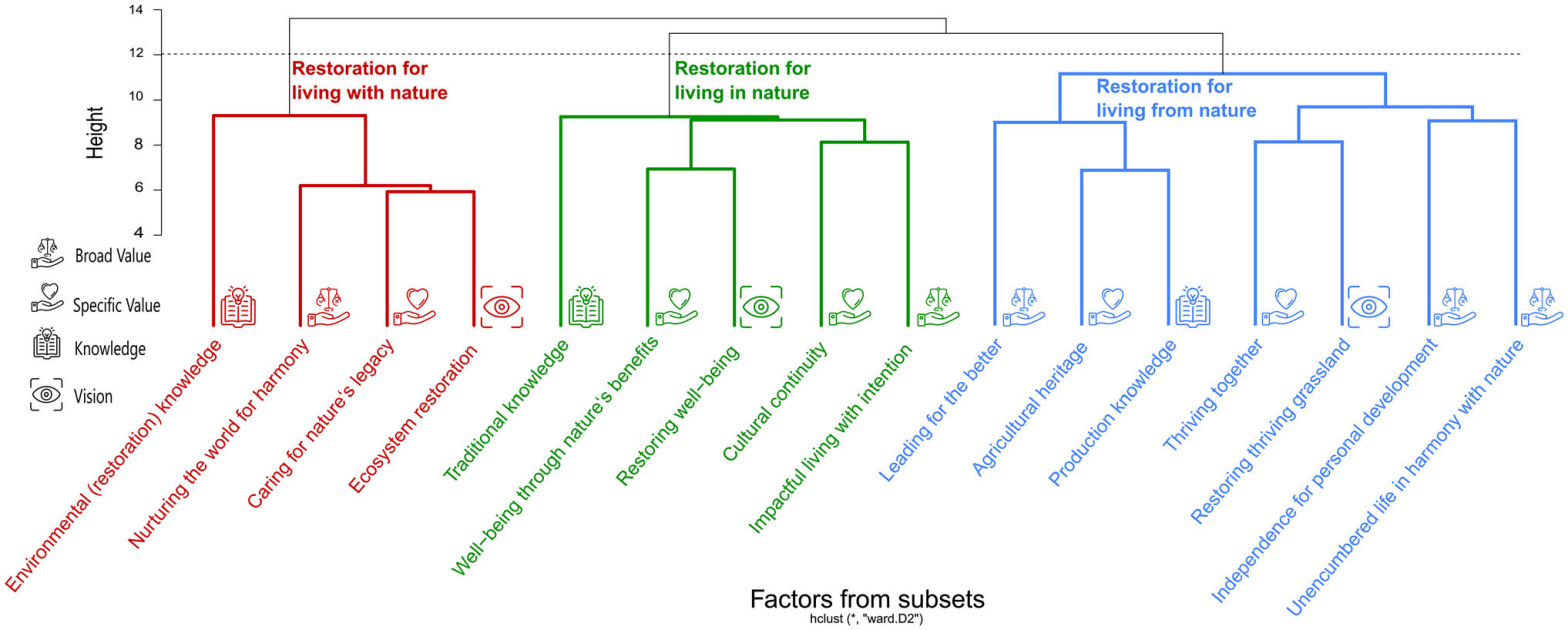
Dendrogram from the cluster analysis of 16 varimax-rotated factors derived from PCA of participants’ broad values, specific values, knowledge and visions from two real-world laboratories for grassland restoration in Germany. Icons from Adobe Stock

Our analysis revealed three potential clusters each with at least one varimax factor from each subset (Fig. 3). The first cluster (red cluster on the left in Fig. 3) consisted of one varimax factor from each subset: “Nurturing the world for harmony” (broad value factor), “caring for nature legacy” (specific value factor), “environmental (restoration) knowledge” (knowledge factor), and “ecosystem restoration” (visions factor). This restoration cluster aligns with the “living with nature” of the Life Framework (O’Connor and Kenter 2019). The environment is the focal point in this cluster, with the knowledge and visions factor centered around ecological properties and values corresponding to conserving and caring for nature, reflecting notions of an ecocentric perspective. We labeled this cluster as **restoration for living with nature: using environmental expertise to revitalize and harmonize with nature**.

The second cluster (green cluster in the middle in Fig. 3) represented anthropocentric properties with knowledge and visions associated with traditions and well-being. Further, the values entailed in this cluster emphasize human life and culture while embracing nature’s benefits for human well-being. In line with the Life Framework, we labeled it as **restoration for living in nature: well-being rooted in sustaining culture and traditional heritage embedded in nature**. This cluster consisted of one factor from the broad values, knowledge, and visions subset as well as two factors from the specific values’ subset. These factors are “impactful living with intention” (broad value factor), “well-being through nature’s benefits” and “cultural continuity” (specific value factors), “traditional knowledge” (knowledge factor), and “restoring well-being” (visions factor).

The third cluster (blue cluster on the right in Fig. 3) was the largest cluster, consisting of three broad value factors, two specific value factors, one knowledge and one visions factor. The varimax factors comprising the third cluster were “independence for personal development”, “unencumbered life in harmony with nature”, “leading for the better” (broad value factors), “thriving together”, “agricultural heritage” (specific value factors), and “production knowledge” (knowledge factor), as well as “restoring thriving grassland” (visions factor). This cluster revolved around production knowledge and a vision of restoring thriving grasslands. It thus conveyed a notion of economic incentive and agricultural practice. This is also reflected in the specific value factor “agricultural heritage”. However, the broad value factors expanded the perspective also towards benefits from the environment with the factor “unencumbered life in harmony with nature”. Nevertheless, this cluster could be regarded as the cluster with the most agricultural focus for social-ecological restoration existing in the real-world laboratories, hence we labeled it as **restoration for living from nature: sustaining traditions and maintaining quality of life by thriving from nature**.

## Discussion

We applied a multi-layered values perspective to the setting of two real-world laboratories for grassland restoration in Germany. Our analysis of the interrelation of broad and specific values, knowledge, and visions for grassland restoration prior to the restoration intervention revealed three clusters depicting values-based social-ecological restoration perspectives prevalent in the real-world laboratories which aligned with the Life Frameworks of Values (O’Connor and Kenter 2019): (1) *restoration for living with nature: using environmental expertise to revitalize and harmonize with nature*, (2) *restoration for living in nature: well-being rooted in sustaining culture and traditional heritage embedded in nature*, and (3) *restoration for living from nature: sustaining traditions and maintaining quality of life by thriving from nature*. Capturing the multi-layered values these three perspectives illustrate potential motivations for engagement in restoration among the participants of the real-world laboratories. Therefore, these perspectives require consideration without jeopardizing one perspective over the others to provide a basis for inclusive (i.e., just and equitable) restoration activities among diverse local actors.

### Values-based social-ecological restoration to embrace nuanced layers

The revealed perspectives align partly with our expectations on how different values relate with knowledge and visions for grassland restoration, yet provide a more nuanced understanding. The *restoration for living with nature* cluster supported our expectation that more intrinsic, biospheric values co-occur with ecological knowledge and visions for ecological restoration. Arguably, this cluster represents a historical ecological restoration approach, which emerged from the “green movement” and emphasizes ecological knowledge to pursue restoration activities (Bradshaw 1993, p. 71). The *restoration for living in nature* with the occurrence of traditional knowledge, cultural values, and a focus on well-being could be perceived as the anthropocentric counterpart of the *restoration for living with nature* cluster. The *restoration for living from nature* cluster did not entirely represent our expectation that values concerning self-enhancement and instrumental values would cluster more with knowledge on forage productivity with visions of restoring grasslands to enhance their capacities to provide specific or short-term benefits. Although the cluster showed the economic and agricultural perspective, it also incorporated the notion of an “unencumbered life in harmony with nature” (broad values factor, Table 5). Under a materialistic and utilitarian understanding of an agricultural economy, this appears incongruent with the agro-economic focus of the cluster. However, the predominant conceptualization of economic practices based on commodification and utilization can be challenged, thus nuancing other values than instrumental underlying economic practices (Ortiz-Przychodzka et al. 2023; Chapman and Deplazes-Zemp 2024). Further, in accordance with O’Connor and Kenter (2019) the *living from nature* frame also recognizes non-material contributions as long as they underpin livelihoods, which is represented in our *restoration for living from nature* cluster. The three clusters indicate overlaps to notions of ecocentrism and anthropocentrism, yet provide more nuances.

Anthropocentrism and ecocentrism have been discussed in environmental ethics and philosophy for an extended period (e.g., Stenmark 2002; Hull et al. 2003) and are also acknowledged within the IPBES values typology (Pascual et al. 2023) as well as in former literature on ecological restoration (Hertog and Turnhout 2018). They can be conceptualized as two ends of a spectrum defining human-nature relationships (Bogert et al. 2022). Our clusters show more intricate and interlinked dimensions of human-nature relationships combining broad values, specific values, knowledge and visions. Expressed also by the Life Framework (O’Connor and Kenter 2019; Kenter and O’Connor 2022) the different value types (i.e., broad and specific values) as well as their categories (e.g., instrumental, intrinsic, and relation values) are straddled across the three different clusters. Although the contrasting human-nature relationships of anthropocentrism and ecocentrism are somewhat represented within the clusters, they convey a more overlapping perspective adding more facets to the simplified spectrum view. The identified clusters could also be perceived as refined views on social-ecological restoration held by participants of the real-world laboratories. This aligns with findings by Sandbrook et al. (2019), who revealed three conservationists’ views: people-centered conservation, science-led ecocentrism, and conservation through capitalism, the latter presenting economic arguments for conservation. All three views find support among conservationists without falling into distinct groups. Similarly, the here emerged clusters present values-based social-ecological restoration perspectives that may be supported to varying degrees across the different participants of the real-world laboratories. Meaning that the perspectives do not necessarily represent distinct groups of actors, but rather actors may relate throughout multiple perspectives. Hence, these perspectives could help to find common grounds and provide diverse reasons for local actors to engage in the restoration process.

### The value of different values-based social-ecological perspectives in grassland restoration

The three perspectives—*restoration for…* (1) *living with nature, (2) living in nature, and (3) living from nature*—illustrate potential motivations for engagement in grassland restoration. Grasslands rely on human interventions, i.e., management through extensive mowing and/or grazing is needed in order to maintain species richness. Therefore, the biggest threats for grassland ecosystems in Central Europe are land abandonment and agricultural intensification (Dengler et al. 2014). Restoration efforts in Europe are often dominated by perspectives focusing on NCP and human well-being, overshadowing the potential to achieve shared benefits for people and nature (Quintero-Uribe et al. 2022). Accordingly, earlier discussions have called for more inclusive restoration initiatives (Elias et al. 2021). Thus, relying only on one values-based social-ecological perspective for restoration interventions will most likely not sustainably improve grasslands. Approaching grassland restoration solely from the perspective of *living with nature* might overlook the perspectives of local actors, thereby reducing the engagement of particular social groups and constraining the potential for widespread stewardship. A dominant focus on *living in nature*, on the other hand, might overlook delicate ecological dynamics and fails to acquire the adequate ecological knowledge to implement restoration measures successfully. A strong *living from nature* approach could emphasize biomass production and run the risk of leaning towards agricultural intensification, jeopardizing restoration projects and extensive grassland management (Kleijn et al. 2008). On that account, recognition and consideration of all three perspectives for grassland restoration in order to cultivate stewardship among the diverse local actors and achieve a common understanding seems to be a promising strategy.

However, despite the potential benefits of acknowledging the multi-layered values perspectives in grassland restoration, our approach lacked direct involvement of restoration critics. The context of this study is most likely relevant to the construction of the perspectives because we targeted those local actors participating in two real-world laboratories for social-ecological grassland restoration. This potentially resulted in responses from people who are either already involved in restoration or are interested in getting involved. This might have influenced the visions’ factors, because in our study approach, we did not include the option to have no desired outcome of the social-ecological restoration, i.e., “no vision”. A “no vision” for restoration could be ascribed to actors who believe they have no stake in healthy ecosystems which aligns with the business-as-usual scenario discussed in archetypal futures literature (Van Vuuren et al. 2012; IPBES 2016; Sitas et al. 2019; Quintero-Uribe et al. 2022). This business-as-usual perspective could play an important and influential role in a real-world laboratory, meaning that some people may have no motivation for getting engaged in restoration at all.

Nevertheless, the three perspectives provide a baseline for exploring how stewardship could be targeted in the real-world laboratories to establish and maintain species-rich grasslands. Our values-based social-ecological perspectives advance discussions about the values and goals of restoration concerning cultural landscapes (Hertog and Turnhout 2018). Acknowledging these different perspectives and their underlying values can help shift restoration towards more inclusive approaches, similar to the calls for more inclusive conservation (Tallis and Lubchenco 2014). As described by Chaplin-Kramer et al. (2023), our approach can depict a first value-centered transformative step by recognizing the multi-layered values of the local people who are part of the restoration context. This understanding could promote the design of transdisciplinary restoration interventions that cultivate stewardship among different actors, resulting perhaps in more inclusive restoration efforts.

### Outlook of transdisciplinarity to cultivate stewardship across the three perspectives

Considering multi-layered values in the realization of inclusive restoration also holds potential for tensions that arise when diverse groups follow distinct agendas (Raymond et al. 2022). To avoid jeopardizing the inclusion of all three perspectives by favoring one over the others, transdisciplinary approaches could accompany the restoration process. Earlier studies have evaluated transdisciplinary research processes for their benefits of social learning and knowledge co-production in regard to achieving sustainable landscapes (Angelstam et al. 2013; Axelsson et al. 2013). Further, Cebrián-Piqueras et al. (2023) have highlighted education, capacity building, and development of partnerships, dialogue and trust, among others, as influential dimensions of inclusive conservation. These dimensions can be targeted through transdisciplinary approaches, such as real-world laboratories (Schäpke et al. 2024), which may pave the way for more inclusive restoration. Further restoration interventions could promote the engagement of multiple actors across the three identified perspectives—*restoration for…* (1) *living with nature, (2) living in nature, and (3) living from nature*—by following a transdisciplinary approach. Future transdisciplinary approaches to social-ecological restoration could also investigate pathways to advance from our current values-based approach towards one aligned with sustainability goals. For example, the fourth Life Frame *living as nature* (O’Connor and Kenter 2019) did not emerge in our study. On the one hand, this absence might stem from the research being conducted in Germany, a highly industrialized, Western nation, where it could be argued that the perspective of *living as nature* is non-existent or only marginally present (e.g., Bogert et al. 2022). On the other hand, it could also reflect the limitations of our survey to capture the perspective of *living as nature*, as highlighted by discussions on how valuation methods frame values expression (e.g., Vatn 2009; Kuhn et al. 2025). We operationalized the IPBES values typology for our approach by adapting survey statements from existing literature. However, this adaptation, combined with the complexity of the typology, may have hindered our ability to capture all perspectives within the Life Framework of Values. Our aim was not to develop a quantitative scale to capture multi-layered values in social-ecological contexts, as this would require a substantial research effort beyond the scope of our study. Instead, our focus was to gain a deeper understanding of the context of potential engagement for grassland restoration within two real-world laboratories. Future research could explore means of operationalizing quantitative and qualitative methodologies to capture the multiple layers of the IPBES values typology and to advance empirical evidence of the Life Frames. Additionally, research should explore if and how transdisciplinary approaches to social-ecological restoration could target the *living as nature* perspective, especially in social-ecological contexts where this perspective is perhaps not present (i.e., Western, industrialized nations) in order to contribute in overcoming the human-nature divide (Mace 2014; West et al. 2020; Artmann 2023; Ghijselinck 2023; Lengieza et al. 2023; Lambert 2024).

## Conclusion

Our application of a multi-layered values assessment, adapted from the IPBES values typology in the context of grassland restoration, revealed three values-based social-ecological restoration perspectives from two real-world laboratories in Germany: (1) *restoration for living with nature: using environmental expertise to revitalize and harmonize with nature*, (2) *restoration for living in nature: well-being rooted in sustaining culture and traditional heritage embedded in nature*, and (3) *restoration for living from nature: sustaining traditions and maintaining quality of life by thriving from nature*. They show not only how the social-ecological restoration context can be perceived in these real-world laboratories, but also how important it is to consider these three different perspectives to enable sustainable grassland restoration. Most species-rich grasslands nowadays rely on human interventions, thus restoration should account for diverse perspectives underlined by multi-layered values without jeopardizing one perspective over others. Moreover, this provides a basis for inclusive (i.e., equitable) restoration that can contribute to cultivating stewardship by different social actors. Further transdisciplinary research for values-based social-ecological restoration is recommended to investigate how to mobilize diverse stewardship perspectives and navigate potential conflicts arising from multi-layered values for the purpose of reversing biodiversity decline and facilitating opportunities for humans to reconnect with nature.

## Supplementary Information

Below is the link to the electronic supplementary material.

Supplementary file1 (PDF 272 KB)

## Data Availability

The dataset for this study is available at the public repository Zenodo and can be accessed at: 10.5281/zenodo.15729357.
