# Supplementary material for: A multi-layered values-based approach to advance social-ecological restoration: Insights from real-world laboratories in Germany
Source: Ambio. 2025 Oct 11;55(3):571–90. doi: 10.1007/s13280-025-02259-w (PMC12868395; doi:10.1007/s13280-025-02259-w)
Supplement: Supplementary file 1 — Supplementary file1 (PDF 272 KB) [file 13280_2025_2259_MOESM1_ESM.pdf]

Ambio

Supplementary Information
This supplementary information has not been peer reviewed

Title: **A multi-layered values-based approach to advance social-ecological restoration: insights from real-world laboratories in Germany**

Contents

Appendix R Script……………………………………………………………………………………………………1

Appendix Histogram of specific values……………………………………………………………………6

Appendix R Script

This is a step-wise description of the analysis performed with R (Version 4.2.3) in R Studio (RStudio 2023.03.0+386 "Cherry Blossom").

R packages

```
library(VIM)
library(psych)
```

Creating data subsets

```
sp_values_na <- subset(data, select=c(2:23))
br_values_na <- subset(data, select = c(45:60))
knowledge_na <- subset(data, select = c(36:44))
visions_na <- subset(data, select = c(24:35))

#Replace NA with the k-nearest neighbours
#VIM adds columns with the indication which variables were imputed (TRUE/FALSE),
#therefore only the columns with the replaced values are selected
sp.values <- kNN(sp_values_na, k = 5)[,1:22]
br.values <- kNN(br_values_na, k = 5)[,1:16]
knowledge <- kNN(knowledge_na, k = 5)[,1:9]
visions <- kNN(visions_na, k = 5)[,1:12]
```

Testing if data is appropriate for exploratory factor analysis (i.e. PCA) following Field et al. (2012)

Bartlett's test

Data is suitable for PCA, because p.value for all subsets < 0.05.

```
cortest.bartlett(sp.values)
```

```
## R was not square, finding R from data
```

```
## $chisq
## [1] 733.1014
##
```

```
## $p.value
## [1] 1.345722e-53
##
## $df
## [1] 231
```

```
cortest.bartlett(br.values)
```

```
## R was not square, finding R from data
```

```
## $chisq
## [1] 331.3041
##
## $p.value
## [1] 1.091072e-21
##
## $df
## [1] 120
```

```
cortest.bartlett(knowledge)
```

```
## R was not square, finding R from data
```

```
## $chisq
## [1] 150.5172
##
## $p.value
## [1] 5.957972e-16
##
## $df
## [1] 36
```

```
cortest.bartlett(visions)
```

```
## R was not square, finding R from data
```

```
## $chisq
## [1] 463.8646
##
## $p.value
## [1] 4.060651e-61
##
## $df
## [1] 66
```

Kaiser-Meyer-Olkin factor adequacy

Values between .5 & .7 are mediocre, values between .7 & .8 are good; values between .8 & .9 are great; values > .9 are superb

```
KMO(sp.values) # overall 0.75 = good
```

```
## Kaiser-Meyer-Olkin factor adequacy
## Call: KMO(r = sp.values)
## Overall MSA =  0.75
## MSA for each item =
##   RV01   RV10   RV15   RV16 InstV2  IntV1   RV02 InstV1   RV03   RV04   RV05
##   0.68   0.70   0.74   0.74   0.79   0.74   0.67   0.70   0.78   0.78   0.66
##   RV07   RV08  IntV2   RV13 InstV3   RV14   RV12   RV06   RV11  IntV3   RV09
##   0.77   0.90   0.64   0.80   0.72   0.76   0.69   0.77   0.85   0.79   0.76
```

```
KMO(br.values) # overall 0.75 = good
```

```
## Kaiser-Meyer-Olkin factor adequacy
## Call: KMO(r = br.values)
## Overall MSA =  0.75
## MSA for each item =
## BV01 BV02 BV03 BV04 BV05 BV06 BV07 BV08 BV09 BV10 BV11 BV12 BV13 BV14 BV15 BV16
## 0.88 0.81 0.76 0.78 0.82 0.68 0.73 0.71 0.60 0.68 0.82 0.48 0.64 0.78 0.81 0.75
```

```
KMO(knowledge) # overall 0.8 = good
```

```
## Kaiser-Meyer-Olkin factor adequacy
## Call: KMO(r = knowledge)
## Overall MSA =  0.8
## MSA for each item =
## Know1 Know2 Know3 Know4 Know5 Know6 Know7 Know8 Know9
##  0.83  0.88  0.83  0.82  0.80  0.79  0.76  0.77  0.34
```

```
KMO(visions) # overall 0.87 = great
```

```
## Kaiser-Meyer-Olkin factor adequacy
## Call: KMO(r = visions)
## Overall MSA =  0.87
## MSA for each item =
##  Ziel1  Ziel2  Ziel3  Ziel4  Ziel5  Ziel6  Ziel7  Ziel8  Ziel9 Ziel10 Ziel11
##   0.95   0.95   0.87   0.73   0.85   0.76   0.90   0.89   0.82   0.87   0.87
## Ziel12
##   0.90
```

PCA without rotation with Spearman correlation (non-parametric values)

```
pca_sp.values_spearman <- principal(sp.values, nfactor=22, rotate="none",
                                    scores = TRUE, cor="spearman")
pca_br.values_spearman <- principal(br.values, 16, rotate="none", scores = TRUE,
                                    cor="spearman")
```

```r
pca_knowledge_spearman <- principal(knowledge, 9, rotate="none", scores = TRUE,
                                  cor="spearman")
pca_visions_spearman <- principal(visions, 12, rotate="none", scores = TRUE,
                                cor="spearman")
```

Varimax-rotated PCA

```r
pca_sp.values_rotated <- principal(sp.values, nfactor=5, rotate="varimax",
                                   scores = TRUE, cor="spearman")
pca_br.values_rotated <- principal(br.values, 5, rotate="varimax",
                                   scores = TRUE, cor="spearman")
pca_knowledge_rotated <- principal(knowledge, 3, rotate="varimax",
                                   scores = TRUE, cor="spearman")
pca_visions_rotated <- principal(visions, 3, rotate="varimax",
                                 scores = TRUE, cor="spearman")
```

Labeling of the factors

```r
sp.values_factor_names <- c("Cultural Continuity",
                            "Thriving together",
                            "Well-being through natures benefits",
                            "Caring for nature legacy",
                            "Agricultural Heritage")
colnames(pca_sp.values_rotated$scores) <- sp.values_factor_names

br.values_factor_names <- c("Nurturing the world for harmony",
                            "Independence for personal development",
                            "Leading for the better",
                            "Unencumbered life in harmony with nature",
                            "Impactful Living with intention")
colnames(pca_br.values_rotated$scores) <- br.values_factor_names

knowledge_factor_names <- c("Environmental (restoration) knowledge",
                            "Production knowledge",
                            "Traditional knowledge")
colnames(pca_knowledge_rotated$scores) <- knowledge_factor_names

visions_factor_names <- c("Ecosystem Restoration",
                          "Restoring 'well-being'",
                          "Restoring thriving grassland")
colnames(pca_visions_rotated$scores) <- visions_factor_names

factor_names <- c(br.values_factor_names,
                  sp.values_factor_names,
                  knowledge_factor_names,
                  visions_factor_names)
```

Storing varimax-rotated PCA scores in a data frame for cluster analysis

```
PCA <- t(cbind(pca_sp.values_rotated$scores,
               pca_br.values_rotated$scores,
               pca_knowledge_rotated$scores,
               pca_visions_rotated$scores))
```

Cluster analysis

```
pca_dist <- dist(PCA, method = "euclidean")
pca_clust <- hclust(pca_dist, method = "ward.D2")
```

Dendrogram

```
plot(pca_clust , main = "Dendrogram for AHC with PCA Components",
     xlab = "Factors from subsets", cex = 1.5, hang = -1)
rect.hclust(pca_clust, k = 3, border = 2)
```

Reference

Field, A., Miles, J., & Field, Z. (2012). Discovering statistics using R (Repr). Sage.

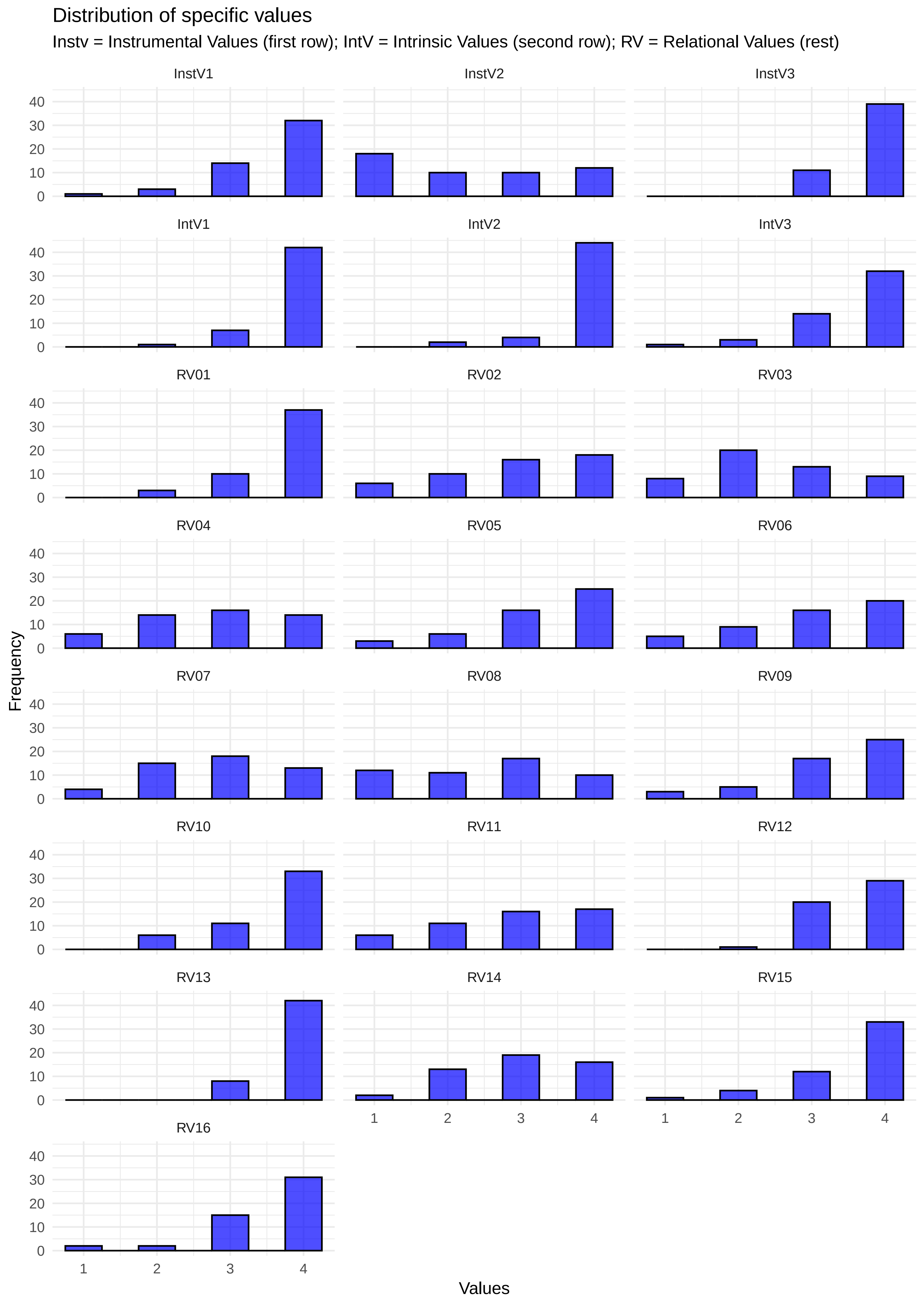

Distribution of specific values
Instv = Instrumental Values (first row); IntV = Intrinsic Values (second row); RV = Relational Values (rest)
InstV1
InstV2
InstV3
IntV1
IntV2
IntV3
RV01
RV02
RV03
RV04
RV05
RV06
RV07
RV08
RV09
RV10
RV11
RV12
RV13
RV14
RV15
RV16
Frequency
Values
0
10
20
30
40
1
2
3
4
